# *tert*-Butyl *N*-{3-[(3-chloro-1,4-dioxo-1,4-dihydro­naphthalen-2-yl)amino]­prop­yl}carbamate

**DOI:** 10.1107/S1600536812029674

**Published:** 2012-07-07

**Authors:** Jackson A. L. C. Resende, Javier A. Gomez

**Affiliations:** a Departmento de Química Inorgânica, Universiade Federal Fluminense, Niterói, CEP 24-020-140, Rio de Janeiro, Brazil

## Abstract

In the title compound, C_18_H_21_ClN_2_O_4_, the mol­ecular sytructure is stabilized by two intra­molecular N—H⋯O hydrogen bonds. In the crystal, mol­ecules are linked by pairs of C—H⋯O hydrogen bonds, forming inversion dimers with graph-set motif *R*_2_^2^(10). N—H⋯O hydrogen bonds further link the dimers into *C*(10) chains along [010].

## Related literature

For biological applications of 2-amino-1,4-naphtho­quinones, see: Kapadia *et al.* (2001 ▶); Brun *et al.* (2005 ▶); Hallak *et al.* (2009 ▶); Bolognesi *et al.* (2008 ▶). For a similar hydrogen-bonding pattern in a related compound, see: Lynch & McClenaghan (2003 ▶). For graph-set notation see: Bernstein *et al.* (1995 ▶).
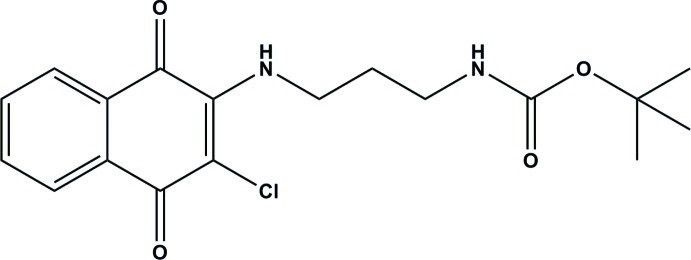


## Experimental

### 

#### Crystal data

C_18_H_21_ClN_2_O_4_
*M* *_r_* = 364.82
Monoclinic, 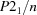

*a* = 5.5172 (2) Å
*b* = 16.6134 (6) Å
*c* = 19.6758 (6) Å
β = 95.709 (3)°
*V* = 1794.53 (11) Å^3^
*Z* = 4
Cu *K*α radiation
μ = 2.10 mm^−1^
*T* = 150 K
0.2 × 0.15 × 0.02 mm

#### Data collection

Agilent Xcalibur Atlas Gemini ultra diffractometer
Absorption correction: multi-scan (*CrysAlis PRO*; Agilent, 2011 ▶) *T*_min_ = 0.551, *T*_max_ = 1
9109 measured reflections
3149 independent reflections
2620 reflections with *I* > 2σ(*I*)
*R*_int_ = 0.040

#### Refinement

*R*[*F* ^2^ > 2σ(*F* ^2^)] = 0.037
*wR*(*F* ^2^) = 0.096
*S* = 1.07
3149 reflections
229 parameters
H-atom parameters constrained
Δρ_max_ = 0.21 e Å^−3^
Δρ_min_ = −0.27 e Å^−3^

### 

Data collection: *CrysAlis PRO* (Agilent, 2011 ▶); cell refinement: *CrysAlis PRO*; data reduction: *CrysAlis PRO*; program(s) used to solve structure: *SHELXS97* (Sheldrick, 2008 ▶); program(s) used to refine structure: *SHELXL97* (Sheldrick, 2008 ▶); molecular graphics: *ORTEP-3 for Windows* (Farrugia, 1997 ▶) and *Mercury* (Macrae *et al.*, 2006 ▶); software used to prepare material for publication: *WinGX* (Farrugia, 1999 ▶).

## Supplementary Material

Crystal structure: contains datablock(s) global, I. DOI: 10.1107/S1600536812029674/bx2415sup1.cif

Structure factors: contains datablock(s) I. DOI: 10.1107/S1600536812029674/bx2415Isup2.hkl

Supplementary material file. DOI: 10.1107/S1600536812029674/bx2415Isup3.cml

Additional supplementary materials:  crystallographic information; 3D view; checkCIF report

## Figures and Tables

**Table 1:** Hydrogen-bond geometry (Å, °)

*D*—H⋯*A*	*D*—H	H⋯*A*	*D*⋯*A*	*D*—H⋯*A*
N1—H1⋯O4	0.85	2.53	3.191 (2)	135
N1—H1⋯O1	0.85	2.1	2.576 (2)	115
N2—H2⋯O2^i^	0.86	2.22	2.873 (2)	132
C8—H8⋯O1^ii^	0.95	2.33	3.200 (2)	153

Symmetry codes: (i) 
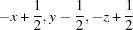
; (ii) 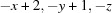.

**Fig. 1.**
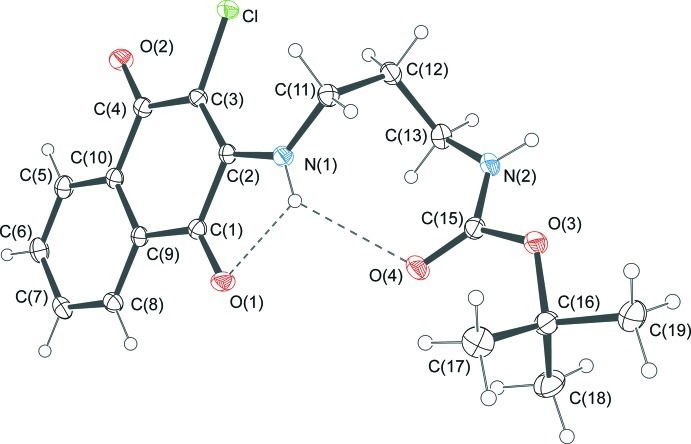
ORTEP representation (Farrugia, 1997) of the molecular structure of compound I with the numbering and displacement ellipsoids at 30% probability level. Hydrogen-bonds are shown by dashed lines.

**Fig. 2.**
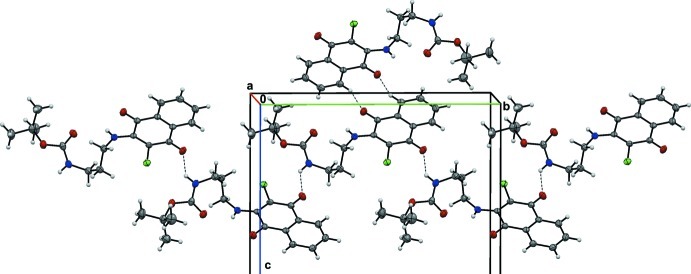
Packing diagram of (I), showing the formation of dimer and chains along [010]. Hydrogen-bonds are shown by dashed lines. "

## supplementary crystallographic information

### Comment

Compounds with the fragment 2-amino-1,4-naphtoquinones shows a variety of uses including antimalarial activity (Kapadia *et al.*, 2001), CDC25 phosphatase inhibitory activity (Brun *et al.*, 2005), antileukemic activity (Hallak *et al.*, 2009), and anticancer potential (Bolognesi *et al.*, 2008). The title compound (I) is the product of the reaction of 2,3-dicloro-1,4-naphtoquionone with *tert-*butyl-3-aminopropylcarbamate. The molecular sytructure is stablibized by a bifurcated hydrogen bond between N atom of one amine group and two O atoms of carbonyl groups N1—H1···O4, N1—H1···O1, like as observed in 2-Chloro-3-(3-dimethylaminopropylamino)-1,4-naphthoquinone (Lynch & McClenaghan, 2003). In the crystal structure the molecules are linked by C—H···O and N—H···O hydrogen bond interactions forming centrosymmetric dimer and chains (along [010]) with graph-set notation R_2_^2^(10) and C(10) respectively (Bernstein, *et al.*, 1995) Table 1, Fig.2

### Experimental

2,3-dichloro-[1,4]-naphthoquinone (2.35 g, 10.34 mmol) was dissolved in acetonitrile (20 ml). Then it was added potassium carbonate (1.43 g, 10.34 mmol) followed by *tert*-butyl-3-aminopropylcarbamate (1.50 g, 8.62 mmol) dissolved in acetonitrile (10 ml). The reaction mixture was refluxed for 5 h and concentrated under reduced pressure. The solution was diluted with ethyl acetate, and washed with saturated sodium carbonate. The organic layer was dried (Na_2_SO_4_) and the solvent was evaporated in vacuum. The residue was purified by column chromatography (silica gel, Hexane/EtOAc, 20:1) to yield *tert-*butyl 3-(3-chloro-1,4-dioxo-1,4-dihydronaphthalen-2-ylamino)propylcarbamate 2.81 g, 89%), mp. 123 – 125 °C. The red crystal compound title were obtained from a solvent mixture (Hexane/EtOAc) *via* slow evaporation. 1H NMR in CDCl3: δ 1.45 (s, 9H, H14), 1.85 (q, J = 6.5 Hz, 2H, H12), 3.25 (q, J = 6.5, 2H, H13), 3.89 (q, J = 6.5 Hz, 2H, H11), 7.61 (td, J = 1.2, 7.6 Hz, 1H, H6/H7), 7.71 (td, J = 1.2, 7.6 Hz, 1H, H6/H7), 8.03 (dd, J = 0.9, 7.6 Hz, 1H, H5/H8), 8.13 (dd, J = 0.9, 7.6 Hz, 1H, H5/H8).

### Refinement

All C-bound H atoms were placed into the calculated idealized positions. The N-bound H atoms were placed at Fourier Maps. All H atoms were refined with fixed individual displacement parameters [*U*_iso_(H) = 1.2Ueq and *U*_iso_(H) = 1.5Ueq(methyl)] using a riding model.

### Crystal data

**Table:**

C_18_H_21_ClN_2_O_4_	*F*(000) = 768
*M**_r_* = 364.82	*D*_x_ = 1.35 Mg m^−^^3^
Monoclinic, *P*2_1_/*n*	Cu *K*α radiation, λ = 1.5418 Å
Hall symbol: -P 2yn	Cell parameters from 3882 reflections
*a* = 5.5172 (2) Å	θ = 3.5–66.1°
*b* = 16.6134 (6) Å	µ = 2.10 mm^−^^1^
*c* = 19.6758 (6) Å	*T* = 150 K
β = 95.709 (3)°	Plate, red
*V* = 1794.53 (11) Å^3^	0.2 × 0.15 × 0.02 mm
*Z* = 4	

### Data collection

**Table:**

Agilent Xcalibur Atlas Gemini ultra diffractometer	3149 independent reflections
Graphite monochromator	2620 reflections with *I* > 2σ(*I*)
Detector resolution: 10.4186 pixels mm^-1^	*R*_int_ = 0.040
ω scans	θ_max_ = 66.3°, θ_min_ = 3.5°
Absorption correction: multi-scan (*CrysAlis PRO*; Agilent, 2011)	*h* = −6→6
*T*_min_ = 0.551, *T*_max_ = 1	*k* = −19→13
9109 measured reflections	*l* = −23→22

### Refinement

**Table:**

Refinement on *F*^2^	Primary atom site location: structure-invariant direct methods
Least-squares matrix: full	Secondary atom site location: difference Fourier map
*R*[*F*^2^ > 2σ(*F*^2^)] = 0.037	Hydrogen site location: inferred from neighbouring sites
*wR*(*F*^2^) = 0.096	H-atom parameters constrained
*S* = 1.07	*w* = 1/[σ^2^(*F*_o_^2^) + (0.0487*P*)^2^ + 0.2773*P*] where *P* = (*F*_o_^2^ + 2*F*_c_^2^)/3
3149 reflections	(Δ/σ)_max_ = 0.001
229 parameters	Δρ_max_ = 0.21 e Å^−^^3^
0 restraints	Δρ_min_ = −0.27 e Å^−^^3^

### Special details

**Table:**

Experimental. CrysAlisPro (Agilent, 2011) Empirical absorption correction using spherical harmonics, implemented in SCALE3 ABSPACK scaling algorithm.
Geometry. All e.s.d.'s (except the e.s.d. in the dihedral angle between two l.s. planes) are estimated using the full covariance matrix. The cell e.s.d.'s are taken into account individually in the estimation of e.s.d.'s in distances, angles and torsion angles; correlations between e.s.d.'s in cell parameters are only used when they are defined by crystal symmetry. An approximate (isotropic) treatment of cell e.s.d.'s is used for estimating e.s.d.'s involving l.s. planes.
Refinement. Refinement of *F*^2^ against ALL reflections. The weighted *R*-factor *wR* and goodness of fit *S* are based on *F*^2^, conventional *R*-factors *R* are based on *F*, with *F* set to zero for negative *F*^2^. The threshold expression of *F*^2^ > σ(*F*^2^) is used only for calculating *R*-factors(gt) *etc*. and is not relevant to the choice of reflections for refinement. *R*-factors based on *F*^2^ are statistically about twice as large as those based on *F*, and *R*- factors based on ALL data will be even larger.

### Fractional atomic coordinates and isotropic or equivalent isotropic displacement parameters (Å^2^)

**Table:**

	*x*	*y*	*z*	*U*_iso_*/*U*_eq_	
Cl	0.06795 (8)	0.53090 (3)	0.20487 (2)	0.02752 (14)	
O2	0.2272 (2)	0.68717 (8)	0.16913 (7)	0.0334 (3)	
O1	0.7377 (2)	0.46056 (8)	0.05404 (7)	0.0309 (3)	
C3	0.2965 (3)	0.54959 (11)	0.15192 (8)	0.0213 (4)	
C2	0.4262 (3)	0.48957 (10)	0.12418 (8)	0.0195 (4)	
C9	0.6701 (3)	0.59958 (11)	0.06765 (8)	0.0215 (4)	
C10	0.5346 (3)	0.65804 (11)	0.09762 (8)	0.0225 (4)	
C5	0.5789 (3)	0.73898 (12)	0.08553 (9)	0.0289 (4)	
H5	0.4862	0.7793	0.1053	0.035*	
C8	0.8508 (3)	0.62166 (12)	0.02638 (9)	0.0254 (4)	
H8	0.9433	0.5815	0.0062	0.03*	
C11	0.2428 (3)	0.35866 (11)	0.16149 (9)	0.0249 (4)	
H11A	0.2312	0.3057	0.1384	0.03*	
H11B	0.079	0.3836	0.156	0.03*	
C4	0.3413 (3)	0.63466 (11)	0.14204 (9)	0.0234 (4)	
C13	0.5714 (4)	0.30943 (12)	0.25169 (10)	0.0295 (4)	
H13A	0.689	0.3429	0.2293	0.035*	
H13B	0.6191	0.3112	0.3015	0.035*	
C1	0.6246 (3)	0.51367 (11)	0.07960 (9)	0.0220 (4)	
C7	0.8947 (3)	0.70260 (12)	0.01492 (9)	0.0292 (4)	
H7	1.0174	0.7181	−0.0131	0.035*	
C12	0.3190 (3)	0.34600 (12)	0.23726 (9)	0.0283 (4)	
H12A	0.3156	0.3985	0.261	0.034*	
H12B	0.1991	0.3103	0.2563	0.034*	
C6	0.7587 (4)	0.76064 (12)	0.04458 (10)	0.0321 (5)	
H6	0.7891	0.816	0.0367	0.039*	
O4	0.7482 (2)	0.25328 (8)	0.12831 (7)	0.0324 (3)	
N2	0.5892 (3)	0.22688 (9)	0.22833 (8)	0.0296 (4)	
H2	0.541	0.189	0.2536	0.036*	
C15	0.6766 (3)	0.20650 (11)	0.16955 (10)	0.0251 (4)	
O3	0.6717 (2)	0.12504 (8)	0.16315 (7)	0.0310 (3)	
C16	0.8079 (3)	0.08385 (12)	0.11316 (10)	0.0299 (4)	
C17	0.6995 (4)	0.10180 (14)	0.04103 (11)	0.0413 (5)	
H17A	0.5265	0.0873	0.0362	0.062*	
H17B	0.7852	0.0704	0.0087	0.062*	
H17C	0.7167	0.1593	0.0316	0.062*	
C18	1.0756 (4)	0.10683 (15)	0.12453 (13)	0.0450 (6)	
H18A	1.0957	0.1629	0.1108	0.067*	
H18B	1.1704	0.0717	0.0971	0.067*	
H18C	1.1332	0.1006	0.173	0.067*	
C19	0.7714 (5)	−0.00400 (13)	0.13100 (13)	0.0472 (6)	
H19A	0.8367	−0.0137	0.1785	0.071*	
H19B	0.857	−0.0382	0.1006	0.071*	
H19C	0.5972	−0.0168	0.1255	0.071*	
N1	0.4113 (3)	0.40938 (9)	0.12852 (7)	0.0242 (3)	
H1	0.524	0.3858	0.1098	0.029*	

### Atomic displacement parameters (Å^2^)

**Table:**

	*U*^11^	*U*^22^	*U*^33^	*U*^12^	*U*^13^	*U*^23^
Cl	0.0286 (2)	0.0288 (3)	0.0266 (2)	0.00320 (18)	0.01000 (17)	0.00058 (18)
O2	0.0422 (8)	0.0250 (7)	0.0349 (7)	0.0059 (6)	0.0136 (6)	−0.0064 (6)
O1	0.0360 (7)	0.0243 (7)	0.0349 (7)	0.0038 (6)	0.0156 (6)	−0.0027 (6)
C3	0.0228 (8)	0.0245 (9)	0.0168 (8)	0.0012 (7)	0.0032 (7)	−0.0004 (7)
C2	0.0218 (8)	0.0215 (9)	0.0147 (8)	−0.0003 (7)	−0.0014 (7)	−0.0004 (7)
C9	0.0232 (8)	0.0233 (9)	0.0173 (8)	0.0001 (7)	−0.0018 (7)	0.0001 (7)
C10	0.0262 (9)	0.0238 (9)	0.0166 (8)	0.0007 (8)	−0.0024 (7)	−0.0016 (7)
C5	0.0349 (10)	0.0229 (10)	0.0280 (10)	0.0024 (8)	−0.0004 (8)	−0.0005 (8)
C8	0.0266 (9)	0.0287 (10)	0.0208 (9)	−0.0012 (8)	0.0023 (7)	0.0002 (8)
C11	0.0250 (9)	0.0222 (9)	0.0272 (10)	−0.0039 (7)	0.0017 (8)	0.0005 (8)
C4	0.0270 (9)	0.0248 (9)	0.0179 (9)	0.0015 (8)	0.0005 (7)	−0.0022 (7)
C13	0.0364 (10)	0.0259 (10)	0.0256 (10)	−0.0009 (8)	0.0006 (8)	0.0013 (8)
C1	0.0242 (8)	0.0227 (9)	0.0186 (8)	0.0017 (7)	−0.0002 (7)	−0.0009 (7)
C7	0.0313 (9)	0.0337 (11)	0.0224 (9)	−0.0064 (8)	0.0010 (8)	0.0049 (8)
C12	0.0340 (10)	0.0252 (10)	0.0264 (10)	−0.0005 (8)	0.0068 (8)	0.0020 (8)
C6	0.0416 (11)	0.0235 (10)	0.0305 (10)	−0.0047 (9)	−0.0006 (9)	0.0044 (8)
O4	0.0372 (7)	0.0274 (7)	0.0341 (7)	−0.0043 (6)	0.0112 (6)	0.0053 (6)
N2	0.0356 (8)	0.0220 (8)	0.0328 (9)	0.0004 (7)	0.0110 (7)	0.0067 (7)
C15	0.0204 (8)	0.0221 (9)	0.0327 (10)	−0.0019 (7)	0.0025 (8)	0.0039 (8)
O3	0.0333 (7)	0.0228 (7)	0.0387 (8)	−0.0013 (6)	0.0131 (6)	0.0023 (6)
C16	0.0260 (9)	0.0262 (10)	0.0380 (11)	0.0019 (8)	0.0061 (8)	0.0010 (9)
C17	0.0432 (12)	0.0420 (13)	0.0382 (12)	−0.0032 (10)	0.0027 (10)	−0.0047 (10)
C18	0.0251 (10)	0.0480 (14)	0.0619 (15)	0.0053 (10)	0.0045 (10)	0.0020 (12)
C19	0.0519 (13)	0.0284 (12)	0.0632 (16)	0.0071 (10)	0.0158 (12)	0.0029 (11)
N1	0.0282 (8)	0.0212 (8)	0.0238 (8)	0.0012 (6)	0.0061 (6)	−0.0006 (6)

### Geometric parameters (Å, º)

**Table:**

Cl—C3	1.7418 (18)	C7—C6	1.386 (3)
O2—C4	1.228 (2)	C7—H7	0.95
O1—C1	1.218 (2)	C12—H12A	0.99
C3—C2	1.372 (2)	C12—H12B	0.99
C3—C4	1.451 (3)	C6—H6	0.95
C2—N1	1.338 (2)	O4—C15	1.218 (2)
C2—C1	1.523 (2)	N2—C15	1.340 (2)
C9—C10	1.392 (2)	N2—H2	0.86
C9—C8	1.396 (3)	C15—O3	1.359 (2)
C9—C1	1.472 (3)	O3—C16	1.465 (2)
C10—C5	1.391 (3)	C16—C17	1.514 (3)
C10—C4	1.496 (3)	C16—C19	1.519 (3)
C5—C6	1.386 (3)	C16—C18	1.520 (3)
C5—H5	0.95	C17—H17A	0.98
C8—C7	1.389 (3)	C17—H17B	0.98
C8—H8	0.95	C17—H17C	0.98
C11—N1	1.454 (2)	C18—H18A	0.98
C11—C12	1.523 (3)	C18—H18B	0.98
C11—H11A	0.99	C18—H18C	0.98
C11—H11B	0.99	C19—H19A	0.98
C13—N2	1.453 (2)	C19—H19B	0.98
C13—C12	1.520 (3)	C19—H19C	0.98
C13—H13A	0.99	N1—H1	0.8486
C13—H13B	0.99		
			
C2—C3—C4	123.52 (16)	C11—C12—H12A	108.9
C2—C3—Cl	123.08 (14)	C13—C12—H12B	108.9
C4—C3—Cl	113.37 (13)	C11—C12—H12B	108.9
N1—C2—C3	131.33 (17)	H12A—C12—H12B	107.7
N1—C2—C1	110.55 (15)	C7—C6—C5	120.86 (18)
C3—C2—C1	118.12 (15)	C7—C6—H6	119.6
C10—C9—C8	120.52 (17)	C5—C6—H6	119.6
C10—C9—C1	120.11 (16)	C15—N2—C13	123.56 (16)
C8—C9—C1	119.37 (17)	C15—N2—H2	118.2
C5—C10—C9	119.41 (17)	C13—N2—H2	118.2
C5—C10—C4	119.90 (17)	O4—C15—N2	125.63 (17)
C9—C10—C4	120.70 (16)	O4—C15—O3	125.34 (18)
C6—C5—C10	119.88 (19)	N2—C15—O3	109.03 (16)
C6—C5—H5	120.1	C15—O3—C16	121.37 (15)
C10—C5—H5	120.1	O3—C16—C17	110.87 (16)
C7—C8—C9	119.66 (18)	O3—C16—C19	101.83 (16)
C7—C8—H8	120.2	C17—C16—C19	110.91 (18)
C9—C8—H8	120.2	O3—C16—C18	109.84 (16)
N1—C11—C12	112.97 (14)	C17—C16—C18	112.07 (18)
N1—C11—H11A	109	C19—C16—C18	110.86 (18)
C12—C11—H11A	109	C16—C17—H17A	109.5
N1—C11—H11B	109	C16—C17—H17B	109.5
C12—C11—H11B	109	H17A—C17—H17B	109.5
H11A—C11—H11B	107.8	C16—C17—H17C	109.5
O2—C4—C3	122.17 (16)	H17A—C17—H17C	109.5
O2—C4—C10	119.68 (16)	H17B—C17—H17C	109.5
C3—C4—C10	118.15 (16)	C16—C18—H18A	109.5
N2—C13—C12	114.05 (16)	C16—C18—H18B	109.5
N2—C13—H13A	108.7	H18A—C18—H18B	109.5
C12—C13—H13A	108.7	C16—C18—H18C	109.5
N2—C13—H13B	108.7	H18A—C18—H18C	109.5
C12—C13—H13B	108.7	H18B—C18—H18C	109.5
H13A—C13—H13B	107.6	C16—C19—H19A	109.5
O1—C1—C9	122.29 (16)	C16—C19—H19B	109.5
O1—C1—C2	118.31 (16)	H19A—C19—H19B	109.5
C9—C1—C2	119.38 (15)	C16—C19—H19C	109.5
C6—C7—C8	119.67 (18)	H19A—C19—H19C	109.5
C6—C7—H7	120.2	H19B—C19—H19C	109.5
C8—C7—H7	120.2	C2—N1—C11	130.65 (16)
C13—C12—C11	113.37 (16)	C2—N1—H1	112.3
C13—C12—H12A	108.9	C11—N1—H1	117
			
C4—C3—C2—N1	179.24 (17)	C10—C9—C1—C2	0.8 (2)
Cl—C3—C2—N1	1.4 (3)	C8—C9—C1—C2	−179.55 (15)
C4—C3—C2—C1	−1.2 (2)	N1—C2—C1—O1	0.8 (2)
Cl—C3—C2—C1	−178.99 (12)	C3—C2—C1—O1	−178.91 (15)
C8—C9—C10—C5	0.7 (2)	N1—C2—C1—C9	179.53 (14)
C1—C9—C10—C5	−179.67 (15)	C3—C2—C1—C9	−0.2 (2)
C8—C9—C10—C4	−179.81 (15)	C9—C8—C7—C6	0.0 (3)
C1—C9—C10—C4	−0.2 (2)	N2—C13—C12—C11	−67.5 (2)
C9—C10—C5—C6	−0.7 (3)	N1—C11—C12—C13	−57.5 (2)
C4—C10—C5—C6	179.74 (16)	C8—C7—C6—C5	−0.1 (3)
C10—C9—C8—C7	−0.3 (2)	C10—C5—C6—C7	0.4 (3)
C1—C9—C8—C7	−179.97 (15)	C12—C13—N2—C15	98.8 (2)
C2—C3—C4—O2	−177.80 (16)	C13—N2—C15—O4	−1.0 (3)
Cl—C3—C4—O2	0.2 (2)	C13—N2—C15—O3	179.19 (15)
C2—C3—C4—C10	1.8 (2)	O4—C15—O3—C16	15.1 (3)
Cl—C3—C4—C10	179.81 (12)	N2—C15—O3—C16	−165.12 (15)
C5—C10—C4—O2	−2.0 (2)	C15—O3—C16—C17	−68.5 (2)
C9—C10—C4—O2	178.51 (15)	C15—O3—C16—C19	173.46 (16)
C5—C10—C4—C3	178.43 (15)	C15—O3—C16—C18	55.9 (2)
C9—C10—C4—C3	−1.1 (2)	C3—C2—N1—C11	3.6 (3)
C10—C9—C1—O1	179.49 (16)	C1—C2—N1—C11	−175.99 (15)
C8—C9—C1—O1	−0.9 (2)	C12—C11—N1—C2	−84.5 (2)

### Hydrogen-bond geometry (Å, º)

**Table:**

*D*—H···*A*	*D*—H	H···*A*	*D*···*A*	*D*—H···*A*
N1—H1···O4	0.85	2.53	3.191 (2)	135
N1—H1···O1	0.85	2.1	2.576 (2)	115
N2—H2···O2^i^	0.86	2.22	2.873 (2)	132
C8—H8···O1^ii^	0.95	2.33	3.200 (2)	153

Symmetry codes: (i) −*x*+1/2, *y*−1/2, −*z*+1/2; (ii) −*x*+2, −*y*+1, −*z*.
